# ENERGY METABOLISM RELATED CANDIDATE GENE ASSOCIATION STUDY OF PERCEIVED PHYSICAL FATIGABILITY

**DOI:** 10.1093/geroni/igac059.1458

**Published:** 2022-12-20

**Authors:** Emma Gay, Adam Santanasto, Ryan Cvejkus, Mary Wojczynski, Mary Feitosa, Nancy W Glynn

**Affiliations:** University of Pittsburgh School of Public Health, Pittsburgh, Pennsylvania, United States; School of Public Health, University of Pittsburgh, Oakmont, Pennsylvania, United States; University of Pittsburgh, Pittsburgh, Pennsylvania, United States; Washington University, Saint Louis, Missouri, United States; Washington University, St Louis, Missouri, United States; School of Public Health, University of Pittsburgh, Pittsburgh, Pennsylvania, United States

## Abstract

Mitochondrial energy production decreases while fatigability increases with age. Genes associated with energy metabolism may contribute to fatigability. Using Long Life Family Study (LLFS), we initially assessed variants (SNPs) in 272 candidate autosomal genes involved in energy metabolism (previously associated with mitochondrial dysfunction disease) with perceived physical fatigability. Two generations of LLFS enrollees (N=2342, mean age=73.7, range 60-108 years) completed the Pittsburgh Fatigability Scale (PFS, 0-50, higher=greater fatigability) at Visit 2 (2014-2017). Physical fatigability prevalence was 42.1% (PFS≥15). Generalized linear mixed models assessed the association of each SNP with continuous PFS (GENESIS R package) adjusted for age, sex, field center, and family relatedness. We found no associations with perceived physical fatigability, all p>2.5E-7 (Bonferroni multiple comparison corrected p-value). Next steps will examine variants in the mitochondrial genome and BTBD3, another promising candidate gene recently discovered. Genetic biomarker(s) may identify individuals susceptible to greater fatigability to target for early intervention.

